# The Association of Meningococcal Disease with Influenza in the United States, 1989–2009

**DOI:** 10.1371/journal.pone.0107486

**Published:** 2014-09-29

**Authors:** Jessica Hartman Jacobs, Cécile Viboud, Eric Tchetgen Tchetgen, Joel Schwartz, Claudia Steiner, Lone Simonsen, Marc Lipsitch

**Affiliations:** 1 Center for Communicable Disease Dynamics, Department of Epidemiology, Harvard School of Public Health, Boston, Massachusetts, United States of America; 2 Division of International Epidemiology and Population Studies, Fogarty International Center, National Institutes of Health, Bethesda, Maryland, United States of America; 3 Department of Biostatistics, Harvard School of Public Health, Boston, Massachusetts, United States of America; 4 Department of Environmental Health, Harvard School of Public Health, Boston, Massachusetts, United States of America; 5 Center for Delivery, Organization, and Markets, Agency for Healthcare Research and Quality, Rockville, Maryland, United States of America; 6 Department of Global Health, School of Public Health and Health Services, George Washington University, Washington, District of Columbia, United States of America; 7 Department of Immunology and Infectious Diseases, Harvard School of Public Health, Boston, Massachusetts, United States of America; University of Cambridge, United Kingdom

## Abstract

**Importance and Objective:**

Prior influenza infection is a risk factor for invasive meningococcal disease. Quantifying the fraction of meningococcal disease attributable to influenza could improve understanding of viral-bacterial interaction and indicate additional health benefits to influenza immunization.

**Design, Setting and Participants:**

A time series analysis of the association of influenza and meningococcal disease using hospitalizations in 9 states from 1989–2009 included in the State Inpatient Databases from the Agency for Healthcare Research and Quality and the proportion of positive influenza tests by subtype reported to the Centers for Disease Control. The model accounts for the autocorrelation of meningococcal disease and influenza between weeks, temporal trends, co-circulating respiratory syncytial virus, and seasonality. The influenza-subtype-attributable fraction was estimated using the model coefficients. We analyzed the synchrony of seasonal peaks in hospitalizations for influenza, respiratory syncytial virus, and meningococcal disease.

**Results and Conclusions:**

In 19 of 20 seasons, influenza peaked≤2 weeks before meningococcal disease, and peaks were highly correlated in time (ρ = 0.95; *P* <.001). H3N2 and H1N1 peaks were highly synchronized with meningococcal disease while pandemic H1N1, B, and respiratory syncytial virus were not. Over 20 years, 12.8% (95% CI, 9.1–15.0) of meningococcal disease can be attributable to influenza in the preceding weeks with H3N2 accounting for 5.2% (95% CI, 3.0–6.5), H1N1 4.3% (95% CI, 2.6–5.6), B 3.0% (95% CI, 0.8–4.9) and pH1N1 0.2% (95% CI, 0–0.4). During the height of influenza season, weekly attributable fractions reach 59%. While vaccination against meningococcal disease is the most important prevention strategy, influenza vaccination could provide further protection, particularly in young children where the meningococcal disease vaccine is not recommended or protective against the most common serogroup.

## Introduction

*Neisseria meningitidis* is a leading cause of meningitis and sepsis in young children and adolescents in the United States, with an estimated incidence of 0.53 cases of invasive meningococcal disease (MD) per 100,00 population, and 1525 cases occurring annually between 1998 and 2007 [1], [2]. It is an important pathogen globally, with incidence estimates varying geographically up to the exceptionally high estimated 1000 cases per 100,000 in the African “meningitis belt” [3].

Reports of MD outbreaks in closed communities following influenza epidemics hint at a possible causal relationship, but direct evidence of antecedent infection with influenza among MD patients is difficult to obtain in most circumstances, though it has been found as a risk factor in certain epidemic settings [4]–[8]. Influenza may no longer be detectable in an individual whose MD was caused by co-infection with influenza, as the virus is rapidly cleared from the nasopharynx within 4 - 10 days of initial symptom onset [9], [10], which is comparable to the incubation period of MD [11], [12]. Evidence of a causal link for influenza predisposing to MD comes from animal studies, disease records of past pandemics, and time series regression models [13]–[17], whose conclusions as with all observational analyses can be considered causal only if confounding factors are adequately accounted for. Previous studies of MD and influenza time series have relied on small numbers of reported MD cases over short time periods and broad categorizations of influenza activity to detect an association between MD and influenza. A study in France over 5 years showed that the incidence of MD in a given week correlated with influenza counts in the preceding 5 weeks and that MD cases were more clinically severe during or up to 2 months after influenza outbreaks [16]. Periods of influenza activity correlated with MD across all age groups in Denmark [17]. A Canadian study provided further evidence using both regression models and a case-crossover design [18].

Influenza could facilitate meningococcal colonization and subsequent invasive disease by several biological mechanisms. Influenza could affect meningococcus transmission by facilitating dispersion of the bacteria or by increasing a person's risk of becoming a carrier when exposed [14]. In mice, influenza-induced immune dysregulation increases susceptibility to invasive MD [19], [20]. Likewise, influenza A neuraminidase increases the adherence of meningococcus to epithelial cells, a necessary step for meningococcus to colonize the nasopharynx [21], [22]. Influenza B, by contrast, does not seem to increase meningococcal adhesion [23].

Given the evidence that influenza infection increases MD risk, we investigated the synchrony of these diseases and quantified the amount of hospitalized MD that is attributable to influenza. This is the largest study to analyze the effects of circulating influenza subtypes, co-circulating respiratory syncytial virus (RSV), and patient age on this association and the only study that quantifies the association using the attributable fraction (AF). We used a large hospitalization database covering 20 influenza seasons in 9 states to explore the role of each of these factors in modifying the fraction of MD attributable to influenza.

## Methods

### Study Population and Data Sources

The study population consisted of all residents in the nine states that continuously contributed data since 1989 to the State Inpatient Databases (SID), a part of the Healthcare Cost and Utilization Project (HCUP) sponsored by the Agency for Healthcare Research and Quality (AHRQ) [24]. Since the investigators of this study had no interaction with patients and received no identifiable private information as part of this study, we were not required to obtain ethics approval or individual patient consent by the Harvard School of Public Health institutional review board under the United States Department of Health and Human Services' regulations on human subjects.

The SID contains all hospitalizations from community hospitals in the participating state and provides the primary and all secondary discharge diagnoses. The nine states (AZ, CA, CO, IA, IL, MA, NJ, WA, WI) were combined to create an aggregate age-stratified dataset by week of admission. We studied the period from January 1, 1989 to November 21, 2009, which represents 20 complete influenza seasons (August 1 through July 31). We removed the final six weeks of 2009 in the dataset to avoid any effects of reporting delays. Working in collaboration with AHRQ, weekly counts of hospitalizations due to MD (*ICD-9-CM* = 036.0-036.9), influenza (*ICD-9-CM* = 487.0-487.9, 488.1) (FLU), or RSV (*ICD-9-CM* = 079.6, 466.11, 480.1) were provided from the SID. We describe our methods for handling missing data in Section S1 of Text S1.

To determine whether influenza subtypes differed in their relationship with MD, we obtained the weekly proportion of positive tests by influenza subtype (B, A/H1N1, A/H3N2 or 2009 pandemic A/H1N1 (pH1N1)) from the Centers for Disease Control and Prevention (CDC) [25]. Testing begins mid-September and ends in May. We used the aggregate national samples to represent the subtype contribution in our nine states, as publicly-available state-level information was not available. Although the relative importance of influenza subtypes can vary somewhat across the United States within a mild season, the most severe seasons (where the putative interaction between influenza and MD would be most salient) are geographically homogeneous (www.cdc.gov/flu). The weekly proportions of positive tests by subtype were multiplied by the weekly count of influenza hospitalizations (FLU_t_) to give a subtype attributable estimate of the weekly number of influenza hospitalizations caused by each subtype (SAIH_t_).

### Analytic Approach

Two sets of analyses were performed to assess the relationship of MD to FLU and RSV. The first assessed the synchrony of timing of peak hospitalizations for MD with FLU and RSV. The second used regression modeling to estimate the fraction of MD attributable to influenza in aggregate, by subtype, patient age, and co-circulating RSV.

#### Synchrony of peak hospitalizations

To compare the timing of the peak in FLU or RSV hospitalizations with MD each season, each time series was smoothed to create a 5-week moving average. If there was a tie for the peak hospitalizations in a season, all tied weeks were included. The synchrony was also explored between influenza subtypes and MD. Given that the subtype-attributable hospitalizations were a product of the viral and hospitalization data, we chose to use only seasons where each subtype was circulating at a meaningful level. This resulted in a season being excluded in the correlation analysis if there were fewer than 75 estimated hospitalizations at the peak for a given subtype. Pearson correlation coefficients were calculated comparing the timing of the peak in each of the 20 seasons.

#### Modeling meningococcal disease

To estimate the fraction of MD attributable to seasonal influenza, we developed a regression model that accounts for the autocorrelation of MD between weeks, the underlying changes in both MD and FLU incidence over time, and seasonality. We have demonstrated that incidence rates of MD using multiple robust datasets has declined substantially since our study period began, while incidence of hospitalizations due to influenza towards the end of our study were higher (Section S2 of Text S1). To account for these changes, MD counts were modeled as a function of season (using sinusoids), trend (using linear and quadratic time terms), autoregressive terms with MD lagged 1–3 weeks, SAIH lagged 1 week, and terms to allow influenza to have a changing effect on MD over time (effect modification).

Unique aspects of our modeling approach, explained further in Section S3 of Text S1, include: the use of a negative binomial generalized linear model with an identity link, autoregressive terms for MD, allowing the amplitude of seasonal forcing to vary annually to reflect changing populations or risk levels, and the use of Legendre polynomials to prevent collinearity of the time covariates [26]. Comparable models were created for MD in 3 age categories as the main outcome (<5, 5–24, and>24 years old) using all age SAIH but for brevity, all further methods will describe the influenza subtype model.

#### Estimating the attributable fraction of meningococcal disease associated with influenza subtypes

We define the AF to be the fraction of hospitalizations caused by MD that could be avoided if influenza infection could be prevented. We estimate the subtype AF based on the coefficients estimated in the model previously described. The numerator of the AF represents the expected MD in a hypothetical influenza-free world while accounting for the autocorrelation of MD generated in the absence of influenza, minus the observed MD incidence in the presence of influenza; thus the numerator is the difference in incidence between a counterfactual influenza-free world and the observed world. To turn this difference into an attributable fraction, it is divided by the observed incidence. The inclusion of autoregressive terms for MD complicates our estimation of the numerator, as we cannot observe what MD would have been in previous weeks in the absence of the effect of influenza. To estimate the AF, we applied the g-formula [27] to generate a chronologically iterative process whereby expected MD counts are estimated using the prior three weeks' estimates for the lagged autoregressive terms. In the first three weeks when we cannot estimate the expected MD count, we use the observed MD. Formally, under the assumption that all common causes of influenza and MD are appropriately accounted for, the g-formula provides an expression for the expected MD counts had one intervened to prevent influenza from occurring in the past. The AF is described further in Section S3 of Text S1.

We varied the possible time lag between influenza and MD from ±7 weeks, including multiple week lags, and chose the model with the best fit as determined by Akaike's Information Criterion. Our use of an additive rather than multiplicative model allowed an unbiased estimate of the cumulative AF when multiple subtypes are included [28] with the result that the overall influenza AF is the sum of the subtype-specific AF.

RSV was initially modeled using the same time lags as FLU, up to 7 weeks before. It was modeled independent of influenza and in models with FLU lagged up to 7 weeks. The best-fitting time lag was 6 weeks for RSV and 1 week for influenza. With these lags, the parameter estimates for RSV were negative (β =  −0.00176) but significant (p-value 0.0009) and the model did not fully converge. As the time lag for RSV decreased, the coefficient became less negative but increasingly also were not significant. After considering the results of both the modeling efforts and peak week analysis, we chose to exclude RSV as a potential contributing factor to MD and removed it from subsequent models.

We estimated 95% confidence intervals of the AF point estimate using a wild bootstrap [29], [30] where each week is randomly assigned a weight from an exponential distribution with a mean of 1 but the chronology and serial correlation between weeks is preserved. The log likelihood in the model is then the product of the weekly weight times the log likelihood for the negative binomial model, which essentially reweights the score equation. For each reweighting, the parameters were estimated by maximum likelihood and an AF for that random weighting scheme was estimated. We generated 1000 independent and identically distributed weights and calculated 1000 AF. The 95% confidence intervals were calculated with the percentile method, as the distributions of AF calculated from the 1000 model runs were not skewed and the mean AF estimate closely approximated the observed AF [31], [32].

In order to assess the impact of unmeasured confounding of the association between MD and influenza by a year-specific or a season-specific common cause, we performed two different permutation tests, described in Section S4 of Text S1.

All statistical analyses were performed using R, Version 2.12.0[33] and the NLMIXED and SURVEYSELECT procedures in SAS® Version 9.3 for Windows XP_PRO.

## Results

In our 20-year study period, the 9 states in the SID recorded 17,575 MD and 242,520 FLU hospitalizations. We attributed 136,813 influenza hospitalizations to H3N2, 42,989 to influenza B, 25,444 to H1N1 and 24,234 to pH1N1. Influenza hospitalizations during months without viral testing were not included (n = 13,040).

**Figure 1:**
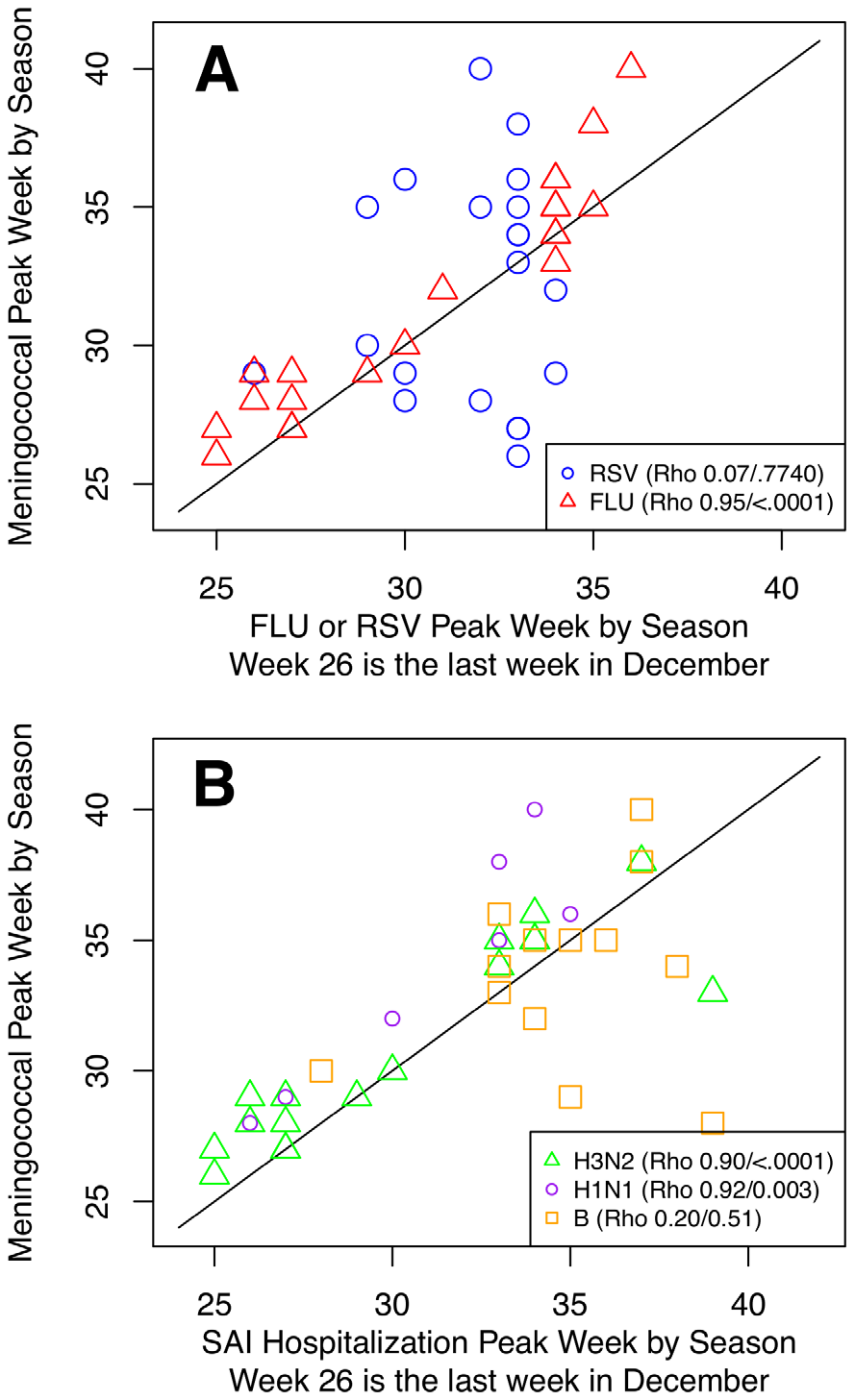
Meningococcal disease (MD) annual peak timing shows strong correlation with peak of influenza (FLU), especially A/H3N2 and A/H1N1 but not RSV. **A** We analyzed 20 seasons and defined the week for each season in which FLU, RSV, or MD had the maximum count as the peak week. The synchrony of the peak week for FLU, RSV, and MD was compared. FLU (marked by a blue triangle) peaked 2 weeks or less before MD in all seasons but one. The peak weeks were highly correlated (ρ = 0.95; *P* <.001). In contrast, RSV (marked by circles) was not synchronized with MD (ρ = 0.07; *P*  = .77) and peaked equally before and after MD. **B **Same analysis repeated for each season and each influenza subtype. Including only seasons with at least 75 subtype-attributable influenza hospitalizations, we analyzed 16 (H3N2), 7 (H1N1), and 13 (B) seasons for correlation with MD. H3N2 (ρ = 0.90; *P* <.001) and H1N1 (ρ = 0.92; *P*  = .003) were highly synchronized with MD hospitalizations while influenza B showed little evidence of an association (ρ = 0.20; *P*  = .51). During our study period, influenza B was the dominant strain in only 2 seasons but in those years peaked with (1990−91) or 1 week before MD (1992−93). The only season when H3N2 or H1N1 peaked after MD was 1992−93 when B dominated.

In the 20 seasons analyzed (1989−1990 to April 15, 2009), the median peak weeks of FLU and MD were weeks 30.5 and 31, respectively (third to fourth week in January). The weeks after April 15, 2009 were treated as a unique season to separate pH1N1 from seasonal influenza. There was no synchrony between MD and pH1N1. This may have been an artifact of using an incomplete year or of the unusual seasonality in FLU that year. The peak in MD for this period was the tail end of the 2008−09 season while FLU peaked in the last week of October, corresponding with the fall wave of pandemic cases, suggesting we needed the full 2009−10 season to observe the MD peak. In all seasons but one, 1992−1993, FLU peaked within 2 weeks before MD; during the 1992−93 season, MD peaked 1 week before FLU (*Figure 1A*). The peak weeks were highly correlated (ρ = 0.95; *P* <.001). This remarkable synchrony of the peak in FLU and MD is observed whether influenza peaks earlier or later in the season. In contrast to influenza, RSV was not synchronized with MD (ρ = 0.07; *P*  = .77) and peaked equally before and after MD.

There was a marked difference in the synchrony of peaks of the different subtype hospitalizations and MD (*Figure 1B*). To look at synchrony only in seasons where each subtype was circulating at a meaningful level, a season was excluded in the correlation analysis if there were fewer than 75 hospitalizations at the peak for a given subtype. This resulted in 16 (H3N2), 7 (H1N1), and 13 (B) seasons analyzed for correlation. H3N2 (ρ = 0.90; *P* <.001) and H1N1 (ρ = 0.92; *P*  = .003) were highly synchronized with MD hospitalizations while influenza B showed little evidence of an association (ρ = 0.20; *P*  = .51). During our study period, influenza B was the dominant strain in only 2 seasons but in those years peaked with (1990−91) or 1 week before MD (1992−93). The only season when H3N2 or H1N1 peaked after MD was 1992−93 when B dominated.

**Figure 2:**
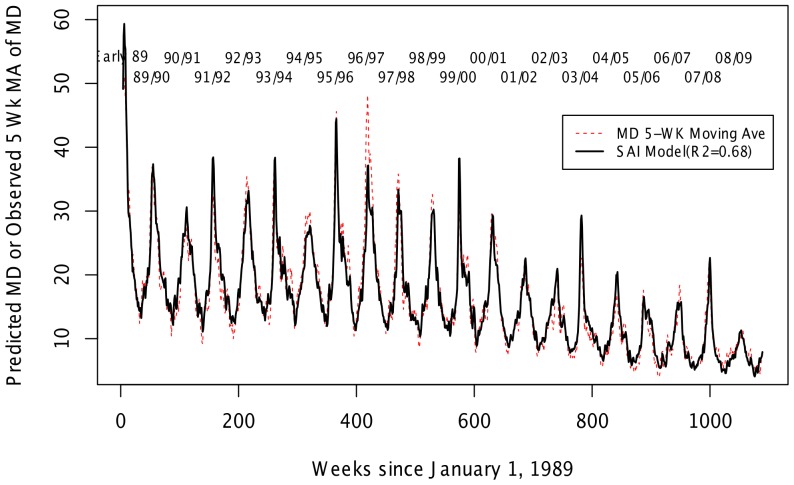
Observed MD compared with model-predicted MD. MD counts were modeled in a negative binomial generalized linear model with an identity link. Independent variables included a sinusoidal term for seasonal variation, linear and quadratic time trends (modeled using Legendre polynomials), autoregressive terms with MD lagged 1−3 weeks, SAIH lagged 1 week, and terms to allow influenza to have a changing effect on MD over time (effect modification).

**Figure 3:**
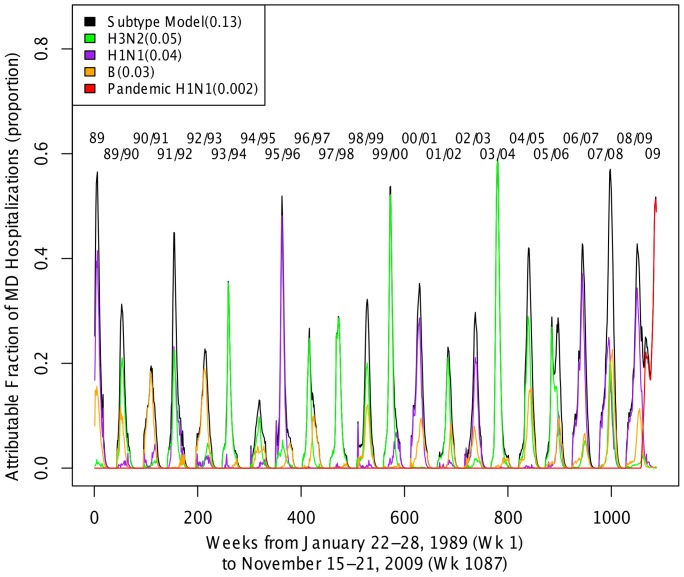
Weekly fraction of MD attributable to all subtypes together and individual influenza subtypes. Most seasons were dominated by either influenza A/H1N1 or A/H3N2, with little of the other subtype. Nearly all seasons also showed a smaller contribution from influenza B.

The model of the association of SAIH and MD explained 68.5% of the variability of MD over 20 years (*Table 1*) and captured the timing of the peaks in MD quite well (*Figure 2*). The model over-predicts MD hospitalizations in the two more severe A/H3N2-dominant influenza seasons. During the 20 years of our study, 12.8% (95% CI, 9.1−15.0) of MD can be attributable to FLU in the preceding weeks with H3N2 accounting for 5.2% (95% CI, 3.0−6.5), H1N1 4.3% (95% CI, 2.6−5.6), B 3.0% (95% CI, 0.8−4.9) and pH1N1 0.2% (95% CI, 0−0.4). During the height of influenza season, AFs reach as high as 59% in a given week for all influenza and H3N2, 48% for H1N1, 23% for influenza B and 51% for pH1N1 (*Figure 3*).

**Table 1:** Modeling results.

Influenza Parameter	Cumulative AF	Model R^2^	Betas	*P* value
	(95% CI)			
All Subtypes Combined	12.8 (9.1−15.0)	68.5%		
H1N1	4.3 (2.6−5.6)		0.002	<.001
H3N2	5.2 (3.0−6.5)		0.0005	<.001
B	3.0 (0.8−4.9)		0.0009	<.001
pH1N1	0.2 (0−0.4)		0.0005	.05

There was little statistical difference between the cumulative AF for each age group, with 12.9 (95% CI, 8.7−15.8), 15.5 (95% CI, 10.6−19.0) and 9.2 (95% CI, 4.9−12.6) percent of the MD attributable to FLU for <5, 5−24, and>25 year olds, respectively. Additional discussion of the results by age and geography can be found in Sections S5 and S6 of Text S1.

After considering the results of both the modeling efforts and peak week analysis, we chose to exclude RSV as a potential contributing factor to MD. The best fitting time lag for RSV was 6 weeks but the RSV parameter estimate was negative and the model did not fully converge (β =  −0.00176; *P* <.001). As the time lag for RSV decreased, the coefficient became less negative but also less significant.

## Discussion

Our study adds to the body of evidence suggesting that influenza infection is a contributing cause of invasive MD, and we have four major findings: 1) the peak in FLU is highly synchronized with the peak in MD hospitalizations; 2) a substantial fraction of MD can be attributed to FLU in the preceding weeks; 3) there is variability in this association by influenza subtype and age, but geography has little to no effect in the areas studied, and 4) RSV is not associated with MD. The remarkable coherence in timing of peaks in influenza and MD hospitalizations over twenty seasons, regardless of when the highly variable influenza season begins, is suggestive of a strong causal relationship. Influenza is neither a necessary nor sufficient cause of MD; however, over a twenty-year period, 13% of MD can be attributed to hospitalizations for seasonal influenza in the preceding weeks. The strength of the causal inference made here, as in any observational study, depends on the adequacy of controls for confounding causes that are associated with the exposure (influenza) and are causally related to the outcome (MD). In particular, we have attempted to control for seasonal or other temporal factors (weather or human behavior, for example) that might be common causes of MD and influenza, through three means: inclusion of sinusoidal and trend terms in the regression, permutation tests to account for uncontrolled seasonal variation, and the use of synchrony analyses that use variation between years in seasonal peak timing to avoid confounding by any factor that shows a consistent seasonal trend.

These findings are consistent with earlier work using time series containing 1–2% as many MD cases as analyzed here [17], [18]. The results of our study differed with previous work on the contribution of RSV and influenza B to MD. Our findings suggest that influenza B contributes less to MD than influenza A but it is still a significant contribution. Others found no association between influenza B and MD but suggested a lack of statistical power as a cause [18]. Our results suggest that RSV is not involved in the causal pathway of developing invasive MD. Two other studies have looked at the association of RSV with MD and have found conflicting evidence. One study supports our results and suggests that RSV is not important in developing MD [34] whereas another study found evidence of an association with RSV [18].

The difference in cumulative AF between the subtypes was not proportional to the relative SAIH contributions to the hospitalization burden over the twenty years; 6∶2∶1∶1 for A/H3N2, B, H1N1 and pH1N1. Since a six-fold higher incidence of H3N2 SAIH exposure does not translate into dramatically higher AF in our study, it suggests that there is not a fixed relationship between the number of FLU hospitalizations and the number of MD hospitalizations, but that this relationship may be modified by influenza subtypes, perhaps the characteristics of circulating meningococcal strains, and age [22], [23], [35]. Using hospitalizations rather than another indicator of influenza activity as our exposure has limitations in that the probability of hospitalization given infection is different among the subtypes [36], [37]. In years with multiple subtypes co-circulating, we likely bias H3N2 towards the null and B and H1N1 away from the null because we assign hospitalizations proportional to the percent of positive tests in a given week regardless of their propensity to cause severe disease necessitating hospitalization. While there is no perfect count of influenza infection in a population, we believe our use of influenza hospitalizations and SAIH is an improvement over solely using viral surveillance data [18], because viral surveillance data alone, expressed as a percentage of positive tests, cannot linearly account for increasing case volume as the influenza season peaks [38], [39]. It has been shown in the UK and US that the percent of tests submitted that were positive for influenza is a poor proxy for influenza morbidity or mortality and poorly reflects the timing and overall severity of an epidemic given changes in viral surveillance over time (both between and within seasons) [40]–[43].

The marginal contribution of pH1N1 to MD in our regression analyses, and lack of temporal synchrony, merits further study with data from 2010 and later. Such analyses may help to distinguish among several hypotheses for the weak or absent association seen here: artifact of the cutoff for data analysis at the end of 2009; different seasonal factors promoting influenza (which departed from the normal seasonality during the pandemic) and MD, swamping the signal of association; or an intrinsic difference in pH1N1's tendency to contribute to MD risk.

The study had several limitations. The ecologic design, using data from 2 independent surveillance systems, makes it impossible to draw direct causal links between influenza and MD. In an ideal study design, we would create a large cohort and follow them prospectively for several years to determine when each person developed influenza or MD. However, the rarity of MD and the difficulty in defining an influenza case would require impossibly large cohorts being sampled frequently for influenza infection.

We also used ICD-9 diagnostic codes from the State Inpatient Database as proxies for infection and disease incidence. These codes are neither perfectly sensitive, as some cases will not be detected, nor perfectly specific, as some cases will be misclassified as FLU or MD when they are not. If the multiplicative relationship between true incidence and each proxy remained constant over the study period, then no error would be introduced by the use of the proxy. Departures from this ideal relationship will add error to our estimates.

We used national information on the relative dominance of each influenza subtype in our regression models, because state-specific data were not available. Surveillance reports however indicate that the most severe A/H3N2 seasons, during which the interaction between influenza and MD is strongest, are spatially homogeneous (eg 1999–2000 or 2003–4) [44], [45]. Further, for our synchrony analyses we used state-specific data on influenza-hospitalization rates to estimate the local incidence of disease activity. As laboratory-based surveillance is strengthened in the US and elsewhere, our models could be improved by inclusion of more local proxies of influenza and RSV virus activity.

Our findings have implications for infectious disease control policy. While vaccination against MD is the most important prevention strategy, vaccination against influenza could provide further protection particularly in the youngest and most vulnerable age group where MD vaccination is not recommended and 13% of MD is attributable to influenza. Given that current MD vaccines do not offer complete protection against all serogroups, including B, one of the three most prevalent causes of invasive disease in the US [46] and the most common in young children [1], immunizing against influenza would result in reductions of MD where MD vaccine cannot. The recent trend toward increasing childhood influenza vaccination in the US may have an impact on MD incidence both through direct protection of influenza-vaccinated persons against influenza which may lead to MD, and through herd immunity to influenza that may offer indirect protection to others against influenza infection leading to MD.

In summary, we found that the age and subtype patterns evidenced in this epidemiological study reinforce the possibility of a biological association between influenza and MD and exclude this association with RSV.

## Supporting Information

Figure S1 **The autocorrelation function of the residuals from a model where the expected count of meningococcal disease in week** ***t*** **is a third order autoregressive process with influenza subtypes lagged 1 week.**
(DOCX)

Figure S2 **Density of calculated attributable fractions from 1,000 bootstrap replicates under the permutation 1 scenario (A) and 10,000 bootstrap replicates under the permutation 2 scenario (B).**
(DOCX)

Figure S3 **Observed 5-week moving average of meningococcal disease (in black) for individual age groups compared with predictions from an autoregressive 3^rd^ order model using influenza subtypes lagged 1 week (in red).**
(DOCX)

Figure S4 **Synchrony in timing of peak hospitalizations for MD and influenza by state.**
(DOCX)

Table S1 **Meningococcal disease hospitalization rates per 100,000 person years by age category in the State Inpatient Database.** Includes 95% confidence intervals and number of patients (n).
(DOCX)

Table S2 **Meningococcal disease hospitalization rates per 100,000 person years by age category in the Active Bacterial Core surveillance system.** Includes 95% confidence intervals and number of patients (n).
(DOCX)

Table S3 **Influenza hospitalization rates per 100,000 person years by age category in the State Inpatient Database.** Includes 95% confidence intervals and number of patients (n).
(DOCX)

Table S4 **Modeling results from age models.**
(DOCX)

Table S5 **Synchrony of meningococcal disease and influenza hospitalizations peak week by state.**
(DOCX)

Text S1 **Supporting information.** Section S1, Missing data. Section S2, Comparing incidence rates of meningococcal disease hospitalizations from multiple data sources. Section S3, Modeling approach. Section S4, Testing for unmeasured confounding. Section S5, The effect of age on the fraction of meningococcal disease attributable to influenza. Section S6. Spatial heterogeneity in the association between influenza and meningococcal disease hospitalizations.
(DOCX)
