# Supplementary material for: The Association of Meningococcal Disease with Influenza in the United States, 1989–2009
Source: PLoS One. 2014 Sep 29;9(9):e107486. doi: 10.1371/journal.pone.0107486 (PMC4180274; doi:10.1371/journal.pone.0107486)

**Figure S1.** The autocorrelation function of the residuals from a model where the expected count of meningococcal disease in week *t* is a third order autoregressive process with influenza subtypes lagged 1 week.


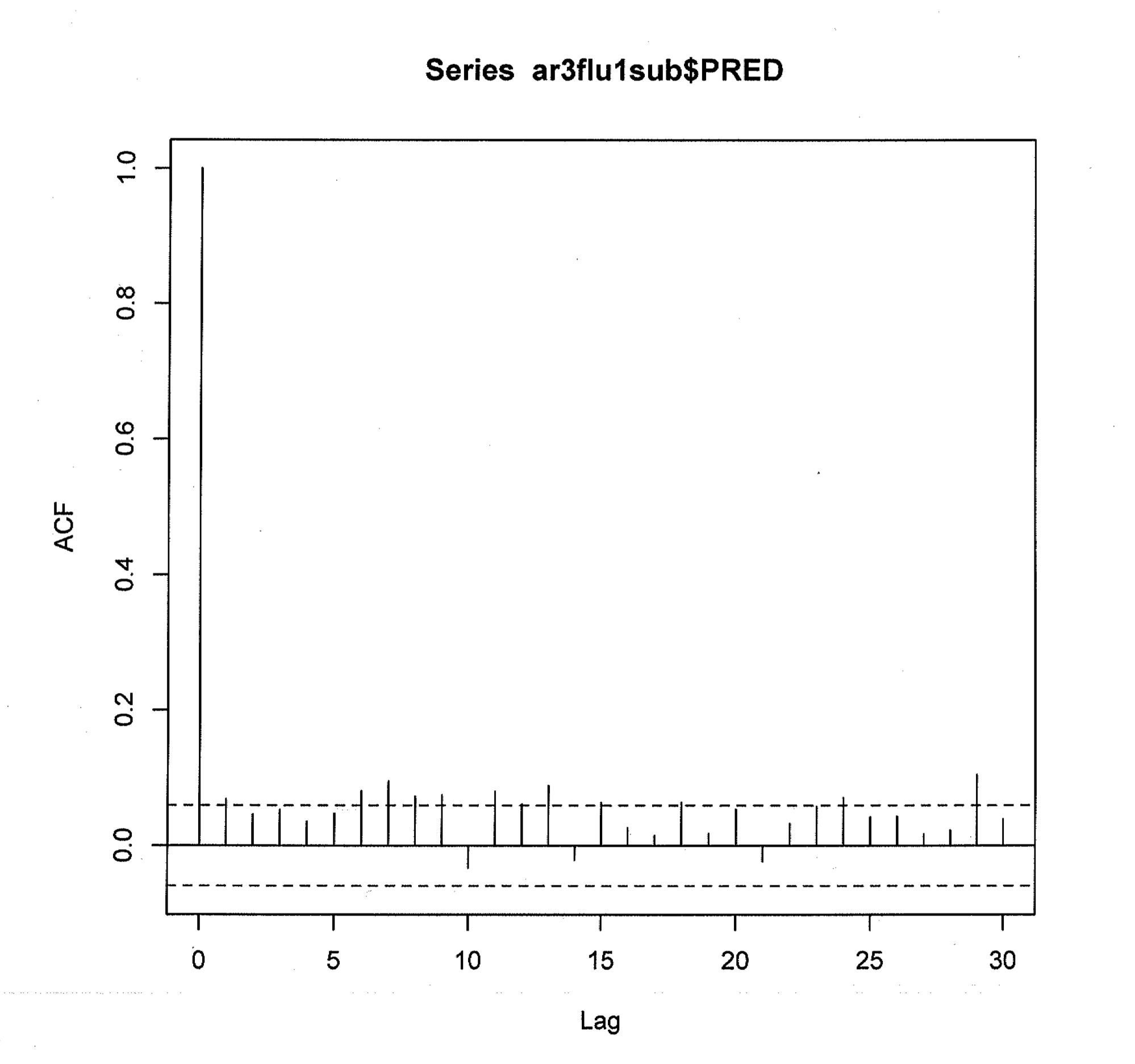

Supplement: Figure S1 — The autocorrelation function of the residuals from a model where the expected count of meningococcal disease in week t is a third order autoregressive process with influenza subtypes lagged 1 week. (DOCX) [file pone.0107486.s001.docx]
