# Supplementary material for: The Association of Meningococcal Disease with Influenza in the United States, 1989–2009
Source: PLoS One. 2014 Sep 29;9(9):e107486. doi: 10.1371/journal.pone.0107486 (PMC4180274; doi:10.1371/journal.pone.0107486)
Supplement: Figure S2 — Density of calculated attributable fractions from 1,000 bootstrap replicates under the permutation 1 scenario (A) and 10,000 bootstrap replicates under the permutation 2 scenario (B). (DOCX) [file pone.0107486.s002.docx]

**Figure S2.** Density of calculated attributable fractions from 1,000 bootstrap replicates under the permutation 1 scenario (A) and 10,000 bootstrap replicates under the permutation 2 scenario (B)
