# Supplementary material for: The Association of Meningococcal Disease with Influenza in the United States, 1989–2009
Source: PLoS One. 2014 Sep 29;9(9):e107486. doi: 10.1371/journal.pone.0107486 (PMC4180274; doi:10.1371/journal.pone.0107486)
Supplement: Figure S3 — Observed 5-week moving average of meningococcal disease (in black) for individual age groups compared with predictions from an autoregressive 3rd order model using influenza subtypes lagged 1 week (in red). (DOCX) [file pone.0107486.s003.docx]

**Figure S3.** Observed 5-week moving average of meningococcal disease (in black) for individual age groups compared with predictions from an autoregressive 3^rd^ order model using influenza subtypes lagged 1 week (in red)
