# Supplementary figures and images for: The Association of Meningococcal Disease with Influenza in the United States, 1989–2009
Source: PLoS One. 2014 Sep 29;9(9):e107486. doi: 10.1371/journal.pone.0107486 (PMC4180274; doi:10.1371/journal.pone.0107486)

**Figure S4.** Synchrony in timing of peak hospitalizations for MD and influenza by state


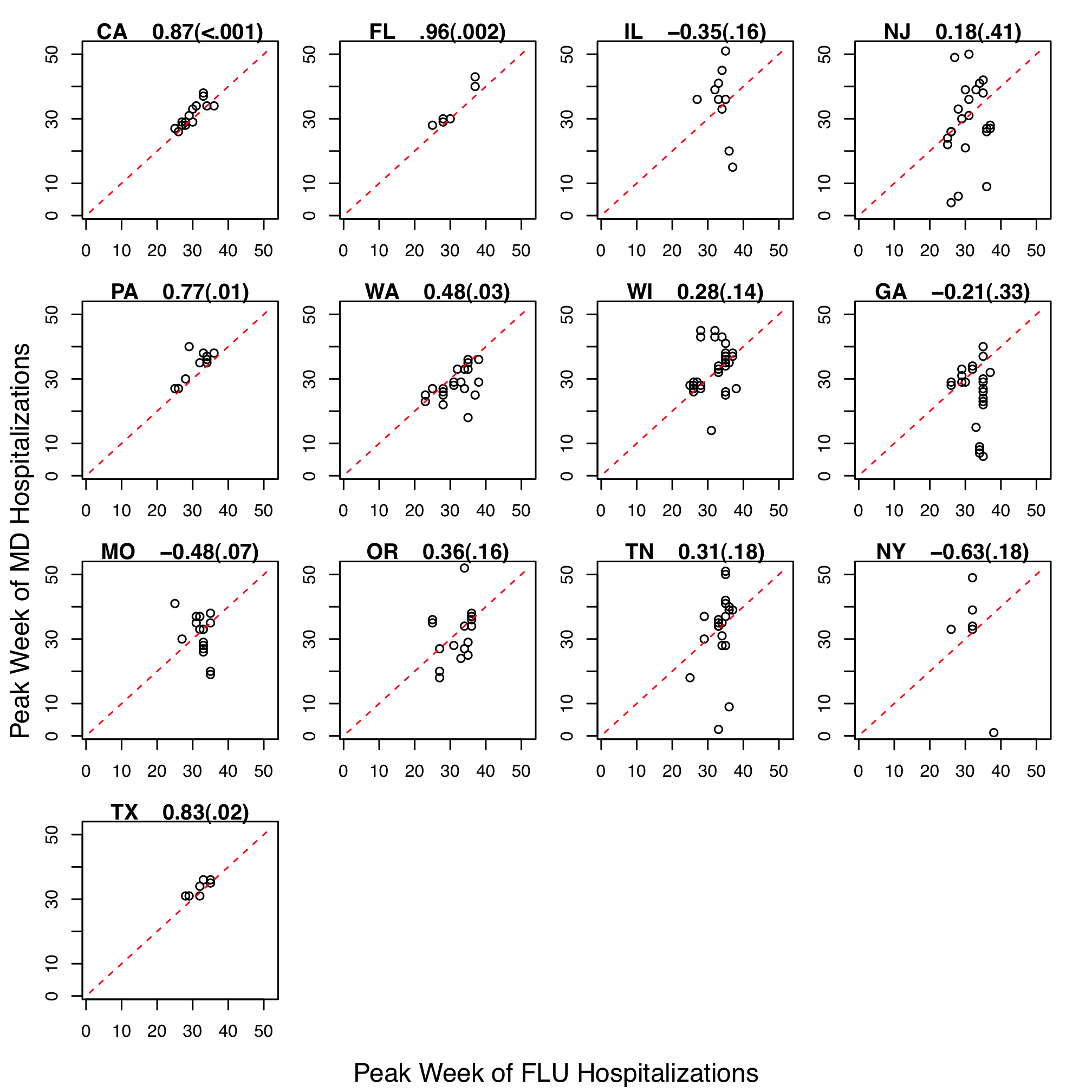

Supplement: Figure S4 — Synchrony in timing of peak hospitalizations for MD and influenza by state. (DOCX) [file pone.0107486.s004.docx]
