# Supplementary material for: The Association of Meningococcal Disease with Influenza in the United States, 1989–2009
Source: PLoS One. 2014 Sep 29;9(9):e107486. doi: 10.1371/journal.pone.0107486 (PMC4180274; doi:10.1371/journal.pone.0107486)
Supplement: Table S1 — Meningococcal disease hospitalization rates per 100,000 person years by age category in the State Inpatient Database. Includes 95% confidence intervals and number of patients (n). (DOCX) [file pone.0107486.s005.docx]

| **Table S1.** Meningococcal disease hospitalization rates per 100,000 person years by age category in the | | | | | | | | | | | | | | | | | |
| --- | --- | --- | --- | --- | --- | --- | --- | --- | --- | --- | --- | --- | --- | --- | --- | --- | --- |
| State Inpatient Database.^a^ Includes 95% confidence intervals and number of patients (n) | | | | | | | | | | | | | | | | | |
|  | **<1 y** | | **1-4 y** | | **5-14 y** | | **15-24 y** | | | | **25-64 y** | | | **>64 y** | | **All Ages** | |
|  |  | **Rates** |  | **Rates** |  | **Rates** | |  | **Rates** |  | | **Rates** |  | | **Rates** |  | **Rates** |
| **Year** | **n** | **95% CI** | **n** | **95% CI** | **n** | **95% CI** | | **n** | **95% CI** | **n** | | **95% CI** | **n** | | **95% CI** | **n** | **95% CI** |
| **1989** | 141 | 23.6 | 166 | 7.8 | 91 | 2.0 | | 70 | 1.4 | 98 | | 0.6 | 41 | | 1.2 | 607 | 1.8 |
|  |  | 19.9-27.9 |  | 6.6-9.0 |  | 1.6-2.4 | |  | 1.1-1.7 |  | | 0.5-0.7 |  | | 0.8-1.6 |  | 1.7-2.0 |
| **1990** | 104 | 15.9 | 138 | 6.1 | 79 | 1.6 | | 64 | 1.3 | 97 | | 0.6 | 34 | | 1.0 | 516 | 1.5 |
|  |  | 13.0-19.3 |  | 5.1-7.2 |  | 1.3-2.0 | |  | 1.0-1.7 |  | | 0.5-0.7 |  | | 0.7-1.3 |  | 1.4-1.7 |
| **1991** | 102 | 15.9 | 112 | 4.7 | 62 | 1.3 | | 60 | 1.2 | 109 | | 0.6 | 33 | | 0.9 | 478 | 1.4 |
|  |  | 13.0-19.3 |  | 3.9-5.7 |  | 1.0-1.6 | |  | 0.9-1.6 |  | | 0.5-0.7 |  | | 0.6-1.3 |  | 1.3-1.5 |
| **1992** | 94 | 15.1 | 110 | 4.5 | 60 | 1.2 | | 87 | 1.8 | 138 | | 0.8 | 33 | | 0.9 | 522 | 1.5 |
|  |  | 12.2-18.5 |  | 3.7-5.4 |  | 0.9-1.5 | |  | 1.4-2.2 |  | | 0.6-0.9 |  | | 0.6-1.3 |  | 1.4-1.6 |
| **1993** | 104 | 17.1 | 128 | 5.1 | 73 | 1.4 | | 80 | 1.7 | 118 | | 0.7 | 42 | | 1.1 | 545 | 1.6 |
|  |  | 14.0-20.8 |  | 4.3-6.1 |  | 1.1-1.8 | |  | 1.3-2.1 |  | | 0.5-0.8 |  | | 0.8-1.5 |  | 1.4-1.7 |
| **1994** | 92 | 15.6 | 104 | 4.2 | 84 | 1.6 | | 88 | 1.9 | 118 | | 0.6 | 36 | | 0.9 | 522 | 1.5 |
|  |  | 12.6-19.1 |  | 3.4-5.1 |  | 1.3-2.0 | |  | 1.5-2.3 |  | | 0.5-0.8 |  | | 0.7-1.3 |  | 1.4-1.6 |
| **1995** | 86 | 14.9 | 124 | 5.1 | 108 | 2.0 | | 87 | 1.8 | 145 | | 0.8 | 60 | | 1.5 | 610 | 1.7 |
|  |  | 11.9-18.4 |  | 4.3-6.1 |  | 1.6-2.4 | |  | 1.5-2.2 |  | | 0.7-0.9 |  | | 1.2-2.0 |  | 1.6-1.9 |
| **1996** | 110 | 14.0 | 161 | 5 | 134 | 1.7 | | 154 | 2.2 | 220 | | 0.8 | 105 | | 1.8 | 884 | 1.7 |
|  |  | 11.5-16.9 |  | 4.3-5.8 |  | 1.5-2.1 | |  | 1.9-2.6 |  | | 0.7-0.9 |  | | 1.5-2.2 |  | 1.6-1.8 |
| **1997** | 130 | 12.9 | 160 | 3.9 | 149 | 1.5 | | 112 | 1.2 | 219 | | 0.6 | 98 | | 1.2 | 868 | 1.3 |
|  |  | 10.8-15.3 |  | 3.4-4.6 |  | 1.3-1.7 | |  | 1.0-1.4 |  | | 0.5-0.7 |  | | 1.0-1.5 |  | 1.2-1.4 |
| **1998** | 130 | 11.7 | 142 | 3.2 | 107 | 1.0 | | 141 | 1.3 | 234 | | 0.6 | 108 | | 1.2 | 862 | 1.1 |
|  |  | 9.7-13.8 |  | 2.7-3.8 |  | 0.8-1.2 | |  | 1.1-1.6 |  | | 0.5-0.7 |  | | 1.0-1.5 |  | 1.0-1.2 |
| **1999** | 96 | 8.6 | 138 | 3.1 | 137 | 1.2 | | 163 | 1.5 | 259 | | 0.6 | 94 | | 1.0 | 887 | 1.1 |
|  |  | 7.0-10.5 |  | 2.6-3.6 |  | 1.0-1.4 | |  | 1.3-1.7 |  | | 0.5-0.7 |  | | 0.8-1.3 |  | 1.0-1.2 |
| **2000** | 90 | 7.4 | 141 | 3.1 | 102 | 0.8 | | 173 | 1.5 | 244 | | 0.6 | 93 | | 1.0 | 843 | 1.0 |
|  |  | 6.0-9.1 |  | 2.6-3.6 |  | 0.7-1.0 | |  | 1.3-1.7 |  | | 0.5-0.6 |  | | 0.8-1.2 |  | 0.9-1.1 |
| **2001** | 87 | 7.1 | 98 | 2.1 | 84 | 0.7 | | 130 | 1.1 | 217 | | 0.5 | 78 | | 0.8 | 694 | 0.8 |
|  |  | 5.7-8.8 |  | 1.7-2.5 |  | 0.5-0.8 | |  | 0.9-1.3 |  | | 0.4-0.5 |  | | 0.6-1.0 |  | 0.7-0.9 |

| **Table S1 continued.** Meningococcal disease hospitalization rates per 100,000 person years by age category in the | | | | | | | | | | | | | | | | | |
| --- | --- | --- | --- | --- | --- | --- | --- | --- | --- | --- | --- | --- | --- | --- | --- | --- | --- |
| State Inpatient Database.^a^  Includes 95% confidence intervals and number of patients (n) | | | | | | | | | | | | | | | | | |
|  | **<1 y** | | **1-4 y** | | **5-14 y** | | **15-24 y** | | | | **25-64 y** | | | **>64 y** | | **All Ages** | |
|  |  | **Rates** |  | **Rates** |  | **Rates** | |  | **Rates** |  | | **Rates** |  | | **Rates** |  | **Rates** |
| **Year** | **n** | **95% CI** | **n** | **95% CI** | **n** | **95% CI** | | **n** | **95% CI** | **n** | | **95% CI** | **n** | | **95% CI** | **n** | **95% CI** |
| **2002** | 78 | 6.3 | 83 | 1.7 | 79 | 0.6 | | 112 | 0.9 | 206 | | 0.4 | 66 | | 0.7 | 624 | 0.7 |
|  |  | 5.0-7.8 |  | 1.4-2.1 |  | 0.5-0.8 | |  | 0.7-1.1 |  | | 0.4-0.5 |  | | 0.5-0.8 |  | 0.7-0.8 |
| **2003** | 70 | 5.6 | 73 | 1.5 | 100 | 0.8 | | 108 | 0.8 | 209 | | 0.4 | 64 | | 0.6 | 624 | 0.7 |
|  |  | 4.3-7.0 |  | 1.2-1.9 |  | 0.6-1.0 | |  | 0.7-1.0 |  | | 0.4-0.5 |  | | 0.5-0.8 |  | 0.7-0.8 |
| **2004** | 59 | 4.8 | 57 | 1.2 | 60 | 0.5 | | 110 | 0.9 | 163 | | 0.3 | 41 | | 0.4 | 490 | 0.6 |
|  |  | 3.6-6.0 |  | 0.9-1.5 |  | 0.4-0.6 | |  | 0.7-1.0 |  | | 0.3-0.4 |  | | 0.3-0.5 |  | 0.5-0.6 |

^a^ Rates calculated from the eight SID States that are also in the ABC Surveillance areas combined: CA, CO, GA, MD, MN, NY, OR, TN. This allowed a comparison between the ABCs rates and those calculated with the SID. Hospitalizations for meningococcal disease were defined as any patient with an ICD-9-CM code = 036.0-036.9 in the discharge diagnosis.
