# Supplementary material for: The Association of Meningococcal Disease with Influenza in the United States, 1989–2009
Source: PLoS One. 2014 Sep 29;9(9):e107486. doi: 10.1371/journal.pone.0107486 (PMC4180274; doi:10.1371/journal.pone.0107486)
Supplement: Table S2 — Meningococcal disease hospitalization rates per 100,000 person years by age category in the Active Bacterial Core surveillance system. Includes 95% confidence intervals and number of patients (n). (DOCX) [file pone.0107486.s006.docx]

| **Table S2.** Meningococcal disease hospitalization rates per 100,000 person years by age category in the Active | | | | | | | | | | | | | | | |
| --- | --- | --- | --- | --- | --- | --- | --- | --- | --- | --- | --- | --- | --- | --- | --- |
| Bacterial Core surveillance system.^a^ Includes 95% confidence intervals and number of patients (n)^b^ | | | | | | | | | | | | | | | |
|  | **<1 y** | | **1-4 y** | | **5-14 y** | | **15-24 y** | | **25-64 y** | | **>64 y** | | **All Age** | | **All Age** |
|  |  | **Rates** |  | **Rates** |  | **Rates** |  | **Rates** |  | **Rates** |  | **Rates** |  | **Rates** | **RD^c^** |
| **Year** | **n** | **95% CI** | **n** | **95% CI** | **n** | **95% CI** | **n** | **95% CI** | **n** | **95% CI** | **n** | **95% CI** | **n** | **95% CI** | **95% CI** |
| **1997** | 39 | 9.4 | 46 | **2.8** | 55 | 1.3 | 55 | 1.4 | 85 | 0.5 | 43 | 1.2 | 323 | **1.1** | **0.2** |
|  |  | 6.7-12.8 |  | **2.0-3.7** |  | 1.0-1.7 |  | 1.0-1.8 |  | 0.4-0.6 |  | 0.9-1.6 |  | **1.0-1.2** | **0.1-0.3** |
| **1998** | 44 | 10.2 | 43 | 2.5 | 44 | 1.0 | 76 | **1.8** | 80 | 0.5 | 43 | 1.2 | 330 | 1.1 | 0.1 |
|  |  | 7.4-13.7 |  | 1.8-3.4 |  | 0.7-1.3 |  | **1.4-2.2** |  | 0.4-0.6 |  | 0.8-1.6 |  | 0.9-1.2 | -0.1-0.2 |
| **1999** | 46 | 10.3 | 32 | **1.8** | 32 | **0.7** | 58 | 1.3 | 75 | **0.4** | 43 | 1.1 | 286 | **0.9** | **0.3** |
|  |  | 7.5-13.7 |  | **1.2-2.5** |  | **0.5-0.9** |  | 1.0-1.7 |  | **0.3-0.5** |  | 0.8-1.5 |  | **0.8-1.0** | **0.1-0.4** |
| **2000** | 29 | 5.7 | 48 | 2.5 | 29 | **0.6** | 62 | 1.3 | 79 | **0.4** | 43 | 1.1 | 290 | **0.8** | **0.2** |
|  |  | 3.8-8.1 |  | 1.8-3.3 |  | **0.4-0.8** |  | 1.0-1.6 |  | **0.3-0.5** |  | 0.8-1.4 |  | **0.7-0.9** | **0.1-0.3** |
| **2001** | 35 | 6.8 | 26 | **1.3** | 30 | 0.6 | 46 | 0.9 | 67 | **0.3** | 26 | 0.6 | 230 | **0.6** | **0.2** |
|  |  | 4.8-9.5 |  | **0.9-1.9** |  | 0.4-0.8 |  | 0.7-1.2 |  | **0.3-0.4** |  | 0.4-0.9 |  | **0.6-0.7** | **0.1-0.3** |
| **2002** | 35 | 7.0 | 23 | 1.2 | 15 | **0.3** | 42 | 0.8 | 60 | 0.3 | 27 | 0.7 | 202 | **0.55** | **0.2** |
|  |  | 4.9-9.7 |  | 0.7-1.7 |  | **0.2-0.5** |  | 0.6-1.1 |  | 0.2-0.4 |  | 0.4-0.9 |  | **0.5-0.6** | **0.1-0.3** |
| **2003** | 22 | 4.0 | 21 | 1.0 | 31 | 0.6 | 42 | 0.8 | 48 | **0.2** | 25 | 0.6 | 189 | **0.5** | **0.2** |
|  |  | 2.5-6.1 |  | 0.6-1.5 |  | 0.4-0.8 |  | 0.6-1.0 |  | **0.2-0.3** |  | 0.4-0.8 |  | **0.4-0.6** | **0.1-0.3** |
| **2004** | 27 | 4.96 | 19 | 0.9 | 19 | 0.4 | 36 | 0.6 | 46 | **0.2** | 16 | 0.4 | 163 | **0.4** | **0.1** |
|  |  | 3.3-7.2 |  | 0.5-1.4 |  | 0.2-0.6 |  | 0.5-0.9 |  | **0.2-0.3** |  | 0.2-0.6 |  | **0.4-0.5** | **0.1-0.2** |
