# Supplementary material for: The Association of Meningococcal Disease with Influenza in the United States, 1989–2009
Source: PLoS One. 2014 Sep 29;9(9):e107486. doi: 10.1371/journal.pone.0107486 (PMC4180274; doi:10.1371/journal.pone.0107486)
Supplement: Table S3 — Influenza hospitalization rates per 100,000 person years by age category in the State Inpatient Database. Includes 95% confidence intervals and number of patients (n). (DOCX) [file pone.0107486.s007.docx]

| **Table S3.** Influenza hospitalization rates per 100,000 person years by age category in the State Inpatient | | | | | | | | | | | | | | |
| --- | --- | --- | --- | --- | --- | --- | --- | --- | --- | --- | --- | --- | --- | --- |
| Database.^a^ Includes 95% confidence intervals and number of patients (n) | | | | | | | | | | | | | | |
|  | **<1 y** | | **1-4 y** | | **5-14 y** | | **15-24 y** | | **25-64 y** | | **>64 y** | | **All Ages** | |
|  |  | **Rates** |  | **Rates** |  | **Rates** |  | **Rates** |  | **Rates** |  | **Rates** |  | **Rates** |
| **Year** | **n** | **95% CI** | **n** | **95% CI** | **n** | **95% CI** | **n** | **95% CI** | **n** | **95% CI** | **n** | **95% CI** | **n** | **95% CI** |
| **1989** | 448 | 36.1  32.8-39.6 | 453 | 9.9  9-10.9 | 467 | 4.5  4.1-4.9 | 823 | 7.4  6.9-7.9 | 3909 | 10.2  9.9-10.5 | 5064 | 57.3  55.7-58.9 | 11164 | 15  14.7-15.3 |
| **1990** | 318 | 24.5  21.8-27.3 | 351 | 7.4  6.6-8.2 | 442 | 4.1  3.7-4.5 | 563 | 5.2  4.8-5.6 | 2357 | 6.1  5.8-6.3 | 2087 | 23.1  22.1-24.1 | 6118 | 8.1  7.9-8.3 |
| **1991** | 455 | 35.4  32.2-38.8 | 479 | 9.8  8.9-10.7 | 511 | 4.7  4.3-5.1 | 785 | 7.3  6.8-7.8 | 3530 | 8.9  8.6-9.2 | 4442 | 48.3  46.9-49.7 | 10202 | 13.3  13.1-13.6 |
| **1992** | 386 | 30.7  27.7-33.9 | 417 | 8.3  7.5-9.2 | 527 | 4.7  4.4-5.2 | 586 | 5.5  5.1-6 | 2515 | 6.3  6-6.5 | 2428 | 25.9  24.9-27 | 6859 | 8.9  8.6-9.1 |
| **1993** | 664 | 53.8  49.7-58 | 492 | 9.7  8.9-10.6 | 403 | 3.6  3.2-3.9 | 660 | 6.2  5.8-6.7 | 3267 | 8.1  7.8-8.4 | 4373 | 46.1  44.8-47.5 | 9859 | 12.6  12.4-12.9 |
| **1994** | 306 | 25.2  22.4-28.2 | 283 | 5.6  5-6.3 | 249 | 2.2  1.9-2.5 | 433 | 4.1  3.7-4.5 | 1918 | 4.7  4.5-4.9 | 1950 | 20.3  19.4-21.2 | 5139 | 6.5  6.3-6.7 |
| **1995** | 568 | 47.7  43.8-51.7 | 456 | 9.2  8.4-10.1 | 404 | 3.4  3.1-3.8 | 469 | 4.4  4-4.8 | 2265 | 5.5  5.3-5.7 | 2209 | 22.7  21.8-23.7 | 6371 | 8  7.8-8.2 |
| **1996** | 696 | 59  54.7-63.6 | 538 | 11.1  10.2-12.1 | 465 | 3.9  3.6-4.3 | 584 | 5.4  5-5.9 | 2810 | 6.7  6.4-6.9 | 3540 | 36.1  34.9-37.3 | 8633 | 10.7  10.5-11 |
| **1997** | 942 | 80.1  75.1-85.4 | 830 | 17.4  16.2-18.6 | 619 | 5.1  4.7-5.6 | 513 | 4.6  4.2-5.1 | 2892 | 6.8  6.6-7.1 | 5258 | 53.3  51.8-54.7 | 11054 | 13.6  13.3-13.8 |
| **1998** | 806 | 68.2  63.5-73 | 659 | 14  12.9-15.1 | 416 | 3.4  3.1-3.8 | 486 | 4.3  3.9-4.7 | 3255 | 7.6  7.3-7.8 | 5085 | 51.2  49.8-52.7 | 10707 | 13  12.8-13.3 |
| **1999** | 1059 | 89.6  84.3-95.2 | 845 | 17.8  16.6-19 | 469 | 3.7  3.4-4.1 | 660 | 5.6  5.2-6 | 5466 | 12.3  11.9-12.6 | 9616 | 96.3  94.4-98.3 | 18115 | 21.3  21-21.6 |
| **2000** | 617 | 49.9  46.1-54 | 556 | 11.8  10.8-12.8 | 500 | 3.9  3.6-4.3 | 411 | 3.4  3.1-3.8 | 1887 | 4.2  4-4.4 | 1389 | 13.8  13.1-14.6 | 5360 | 6.2  6.1-6.4 |
| **2001** | 1052 | 85.9  80.8-91.3 | 1071 | 22.5  21.1-23.8 | 513 | 4  3.7-4.4 | 385 | 3.1  2.8-3.5 | 2003 | 4.4  4.2-4.6 | 2800 | 27.6  26.6-28.7 | 7824 | 9  8.8-9.2 |
| **2002** | 699 | 56.2  52.1-60.5 | 672 | 14  12.9-15.1 | 496 | 3.9  3.6-4.3 | 323 | 2.6  2.3-2.9 | 1277 | 2.8  2.6-2.9 | 1056 | 10.3  9.7-10.9 | 4523 | 5.2  5-5.3 |
| **2003** | 3326 | 265.1  256.2-274.3 | 3691 | 75.6  73.2-78.1 | 1281 | 10.2  9.6-10.8 | 1116 | 8.9  8.4-9.4 | 4968 | 10.6  10.3-10.9 | 9375 | 90.7  88.8-92.5 | 23757 | 26.9  26.5-27.2 |
| **2004** | 1290 | 103  97.4-108.7 | 1183 | 23.9  22.6-25.4 | 627 | 5.1  4.7-5.5 | 480 | 3.8  3.4-4.1 | 4123 | 8.7  8.4-9 | 8706 | 83.2  81.5-85 | 16409 | 18.4  18.1-18.7 |

^a^ The nine states that have continuously contributed data to the State Inpatient Database (SID) include AZ-CA-CO-IA-IL-MA-NJ-WA-WI. Influenza hospitalizations include any patient with an ICD-9-CM code of 487.0-487.9 anywhere in the discharge diagnosis.
