# Supplementary material for: The Association of Meningococcal Disease with Influenza in the United States, 1989–2009
Source: PLoS One. 2014 Sep 29;9(9):e107486. doi: 10.1371/journal.pone.0107486 (PMC4180274; doi:10.1371/journal.pone.0107486)
Supplement: Table S4 — Modeling results from age models. (DOCX) [file pone.0107486.s008.docx]

| **Table S4.** Modeling results from age models | | |  |  |  |
| --- | --- | --- | --- | --- | --- |
|  |  | **Cumulative AF** |  | **Parameter** |  |
| **Age, y** | **Influenza Parameter** | **(95% CI)** | **Model R^2^** | **estimate** | ***P* value** |
| 0-4 | All Subtypes Combined | 12.9 (8.7-15.8) | 40.7% |  |  |
|  | H1N1 | 4.3 (2.5-5.6) |  | 0.0023 | <.001 |
|  | H3N2 | 5.4 (3.1-6.9) |  | 0.0006 | <.001 |
|  | B | 3.3 (0.6-6.0) |  | 0.0009 | .03 |
|  | pH1N1 | 0.01 (-0.2-0.2) |  | 0.00002 | .92 |
| 5-24 | All Subtypes Combined | 15.5 (10.6-19.0) | 30.7% |  | <.001 |
|  | H1N1 | 4.1 (1.8-6.4) |  | 0.0026 | <.001 |
|  | H3N2 | 6.4 (3.2-8.4) |  | 0.0006 | <.001 |
|  | B | 4.5 (1.4-7.1) |  | 0.0015 | .005 |
|  | pH1N1 | 0.5 (0.01-0.8) |  | 0.0032 | .20 |
| >24 | All Subtypes Combined | 9.2 (4.9-12.6) | 23.4% |  |  |
|  | H1N1 | 3.3 (1.3-5.0) |  | 0.0017 | <.001 |
|  | H3N2 | 3.7 (1.2-5.7) |  | 0.0003 | <.001 |
|  | B | 1.9 (-1.0-1.9) |  | 0.0006 | .15 |
|  | pH1N1 | 0.3 (-0.1-0.6) |  | 0.0003 | .19 |
