# Supplementary material for: The Association of Meningococcal Disease with Influenza in the United States, 1989–2009
Source: PLoS One. 2014 Sep 29;9(9):e107486. doi: 10.1371/journal.pone.0107486 (PMC4180274; doi:10.1371/journal.pone.0107486)
Supplement: Table S5 — Synchrony of meningococcal disease and influenza hospitalizations peak week by state. (DOCX) [file pone.0107486.s009.docx]

| **Table S5.** Synchrony of the peak week of meningococcal disease and influenza hospitalizations by state | | | | |
| --- | --- | --- | --- | --- |
|  | **Correlation coefficient** | **Number of weeks** | **Number of** | **Mean weekly MD cases** |
| **State** | **(*P* value)** | **included^a^** | **seasons included^b^** | **over 20 year period** |
| Wisconsin | 0.28 (.14) | 30 | 18 | 1.5 |
| Tennessee | 0.31 (.18) | 21 | 12 | 1.7 |
| Georgia | -0.21 (.33) | 23 | 11 | 1.8 |
| New Jersey | 0.18 (.41) | 23 | 18 | 2.0 |
| Missouri | -0.48 (.07) | 15 | 10 | 2.2 |
| Oregon | 0.36 (.16) | 17 | 10 | 2.4 |
| Washington | 0.48 (.03) | 20 | 15 | 2.8 |
| New York | -0.63 (.18) | 6 | 5 | 3.3 |
| Illinois | -0.35 (.32) | 10 | 10 | 3.5 |
| Texas | 0.83 (.02) | 7 | 5 | 4.1 |
| Pennsylvania | 0.77 (.01) | 10 | 8 | 5.3 |
| Florida | 0.96 (.002) | 6 | 4 | 7.0 |
| California | 0.87 (<.001) | 16 | 15 | 14.0 |

^a^The number of weeks included in the correlation analysis. All MD weeks that were tied were included. As the average number of weekly MD cases decreases, the number of observations increases because the 5-week moving average results in more tied weeks.

^b^The number of seasons included in the study is influenced both by the time contributed to the SID by the state and the number of years when the peak number of cases was greater than 3.5% of the annual cases in that state.
