# Supplementary material for: The Association of Meningococcal Disease with Influenza in the United States, 1989–2009
Source: PLoS One. 2014 Sep 29;9(9):e107486. doi: 10.1371/journal.pone.0107486 (PMC4180274; doi:10.1371/journal.pone.0107486)
Supplement: Text S1 — Supporting information. Section S1, Missing data. Section S2, Comparing incidence rates of meningococcal disease hospitalizations from multiple data sources. Section S3, Modeling approach. Section S4, Testing for unmeasured confounding. Section S5, The effect of age on the fraction of meningococcal disease attributable to influenza. Section S6. Spatial heterogeneity in the association between influenza and meningococcal disease hospitalizations. (DOCX) [file pone.0107486.s010.docx]

**Text S1 Supporting Information**

**The association of meningococcal disease with influenza in the United States, 1989-2009**

Jessica Hartman Jacobs, Cécile Viboud, Eric Tchetgen Tchetgen, Joel Schwartz, Claudia Steiner, Lone Simonsen, Marc Lipsitch

**Section S1. Missing data**

In order to ensure that cases were not double counted in two time series, which would upwardly bias any association, a hospitalization that had influenza and meningococcal disease [MD] was only counted as a MD hospitalization. Initial examination of the respiratory syncytial virus [RSV] time series revealed an increasing trend in hospitalizations starting in the 1996/1997 winter season that was most likely due to additional *ICD-9-CM* codes for RSV that were added in October of 1996 to the previously singular classification of RSV pneumonia.[^1^](#_ENREF_1) To account for this trend, RSV counts for each state were de-trended by multiplying the weekly hospitalization count before July 1, 1996 by the ratio of the average weekly hospitalizations after and before July 1, 1996.

In the tests that were positive for influenza submitted to the CDC viral surveillance program,[^2^](#_ENREF_2) positive unsubtyped influenza A isolates in a given week were distributed to either influenza A/H1N1, A/H3N2 or 2009 pandemic H1N1 (pH1N1) counts based on the proportion of positive subtyped influenza A tests accounted for by each subtype that week. When there were no positive A/H1N1, A/H3N2 or pH1N1 (only in 2009) but positive A/unsubtyped isolates, the ratio of positive tests from the previous week or the following week was used, with preference given to the previous week. If there were no positive A/H1N1, A/H3N2 or pH1N1 (only in 2009) in the previous or following weeks, the unsubtyped isolates were divided equally between A/H1N1, A/H3N2 and pH1N1 (only in 2009).

**Section S2. Comparing incidence rates of meningococcal disease hospitalizations from multiple data sources**

An initial objective of this study was to determine whether the relationship between influenza and MD varied among the serogroups of MD. We used serogroup specific weekly counts of MD from the Active Bacterial Core surveillance (ABCs)[^3^](#_ENREF_3),[^4^](#_ENREF_4) to approach the problem, but the incidence by serogroup is so small that we could not estimate meaningful models that converged. Of secondary interest was to use the ABCs system to assure that the State Inpatient Database (SID)[^5^](#_ENREF_5) hospitalization data that we did use in our study correspond with nationally published rates of MD. In particular, we were interested in understanding whether the MD hospitalization rates calculated from the SID would be comparable to the MD cases reported to the CDC ABCs program. The ABCs is an ongoing active population- and laboratory- based surveillance system that is part of the Emerging Infections Program Network coordinated by the CDC and it captures all cases of MD, regardless of hospitalization status.

The SID and ABCs cover different time periods, epidemic years, and geographic regions, so we converted census data to person months in order to create a consistent denominator between the populations and to calculate rates for a given influenza or MD season, using August 1^st^ to July 31^st^ of the following year. There are 8 states in the SID database that overlap the ABCs surveillance area. In the SID, states joined at the beginning of the calendar year so only 7 months of person time were accrued for the rate calculation in the matching epidemic year. For example, NY State joined in January 1, 1998, so for the 1997/98 epidemic year, NY contributed only 7 months of person time. Annual population estimates for each state were obtained from the US Census Bureau.[^6^](#_ENREF_6) As inter-censal estimates are made on July 1 of each year, the population estimate was applied to each epidemic year rather than calendar year. In a given epidemic year, the population file used was the epidemic year + 1. For example, in the 1997/1998 season, the 1998 population file was used.

Person time was calculated differently for the 10 states in the ABCs surveillance system, as many states changed which counties were represented in the dataset during the 8 year period and the enrollment was open, so new counties could join at any point in the year. To calculate the person time contributed to each epidemic year, we used two census years of data that were matched to the counties under surveillance. The total person months contributed were then divided by 12 to get person years. The surveillance populations were provided by the ABCs group.

We estimated 95% confidence intervals for all rates using an R function based on a method which uses the gamma distribution to calculate exact confidence intervals for a Poisson count.[^7^](#_ENREF_7)

The rates of MD hospitalizations from the SID decreased dramatically for all ages during this time series. In 1989/90, there was an annual rate of 1.8 (1.7-2.0) hospitalizations per 100,000 people and by 2004/05 the rate was 0.6 (0.5-0.6) (see eTable1). This decreasing trend was dominated by a dramatic decline in rates in children under the age of 15, and most notably under 1 year of age. The age specific and all age rates calculated using the ABCs surveillance data were very similar to the SID rates. A notable decline in rates is also detectable in the ABCs data, particularly in the under 1 year olds. A rate difference and 95% confidence interval was calculated between the ABCs and SID rates and the all age rate difference was reported in eTable2. Using these rates we concluded that the SID represents the full extent of MD in the states used in our study.

The rate of influenza hospitalizations has been increasing over the seventeen year time series, with the highest rates observed in infants under 1 year old and adults over 65 years of age (eTable3). The most severe season for hospitalizations was the 2003/04 influenza season, where rates in all age groups increased dramatically. The most dramatic increase was in infants, who experienced a 2.5 fold increase in incidence over the next highest season, 2004/05. We used these rates to help define our choice in model parameters to best capture the variability between seasons and the additional increasing trend in influenza hospitalizations over our period of study.

**Section S3. Modeling approach**

Our choice in model structure was motivated by the inherent underlying complexity and dependence of infectious disease time series. To model MD and FLU appropriately, we needed to account for long term trends, seasonality, inter-annual variability from changes in population susceptibility and pathogen mutation, and serial correlation between weeks given the infectious nature of both diseases. The weekly counts of MD and FLU were over-dispersed so we employed a generalized linear model with a negative binomial error distribution.[^8^](#_ENREF_8) An identity link function (rather than the more common log link) was used to represent the linear association between influenza and MD based on a mechanistic assumption that the number of MD hospitalizations in a given week will be linearly related to the amount of influenza morbidity in a given week (with the appropriate time lag accounted for) plus the effects of seasonal forcing and any time trends. The seasonal terms were multiplied by the trend terms to reflect a constant degree of seasonal variation reflected in a changing population and/or level of risk. This choice was prompted by the inter-annual variability reflected in the incidence rates in eTables 1 to 3. Time was modeled using first and second order shifted Legendre polynomial transformations to make the linear and quadratic time covariates orthogonal and prevent collinearity.[^9^](#_ENREF_9) Given the variability in dominant subtype and MD serogroup over time and the inverse trends in MD and FLU, we allowed FLU to have a varying effect on MD over time by adding in terms for effect modification (represented by the middle line in the following equation). In order to assess how many weekly autoregressive terms for MD were necessary, we fit models using AR terms with MD lagged 1:4 weeks and plotted the autocorrelation function of the residuals for each model. Autocorrelation between MD weeks was most appropriately accounted for with an AR3 model, where MD at 1, 2 and 3 week lags was included. The following equation describes the model: *t* is the number of weeks since the start of the time series on January 1, 1989 with *t_max_=*1086, η_t_ represents annual variability in seasonal forcing, and *x_t_* is a scaled time variable used to generate the Legendre polynomials that varies from 0 to 1. The *SAIH_S=s, t-1_* term represents the SAIH lagged 1 week for H1N1 (*s*=5), H3N2 (*s*=6), B (*s*=7) or pH1N1 (*s*=8).

$$\begin{matrix} E[MD_{t}]=\beta_{0}\eta_{t}+\beta_{1}\eta_{t}(2x_{t}-1)+\beta_{2}\eta_{t}(6x_{t}^{2}-6x_{t}+1)+ \\ \sum_{s=5}^{8} \left[\beta_{0}\beta_{s}SAIH_{S=s,t-1}+\beta_{1}\beta_{s}SAIH_{S=s,t-1}(2x_{t}-1)+\beta_{2}\beta_{s}SAIH_{S=s,t-1}(6x_{t}^{2}-6x_{t}+1)\right]+ \\ \beta_{9}MD_{t-1}+\beta_{10}MD_{t-2}+\beta_{11}MD_{t-3} \end{matrix}$$

$\eta_{t}=1+\beta_{3}\cos\frac{2\pi t}{52}+\beta_{4}\sin\frac{2\pi t}{52}$

$x_{t}=\frac{t-1}{t_{\max}}$

While the models described so far use SAIH lagged 1 week, we tested lags ranging from -7 weeks to 7 weeks. We chose the time lag (*k*) with the best fit, as determined by Akaike Information Criteria (AIC) using a dataset that truncated the first and last 7 weeks of the time series. We confirmed the appropriateness of the chosen lags by calculating the cross correlation function between the MD and FLU time series and plotted the autocorrelation function of the model residuals to check for residual autocorrelation. eFigure 1 shows the autocorrelation plot for the subtype model with FLU lagged one week, which was the model with the best fit. Once the appropriate time lag was determined for each model, the remaining weeks were added back into the dataset. To describe the proportion of variability that was accounted for by the statistical models, we calculated a pseudo-R^2^. [^8^](#_ENREF_8),[^10^](#_ENREF_10)

For each subtype of influenza, we calculate the fraction of MD attributable to hospitalizations caused by that subtype using the coefficients from the model. In the following equation, we demonstrate the calculation of a single subtype AF, for H1N1 SAIH.

$$AF_{SAIH=H1N1}=1-\left[\frac{\sum_{t} \widehat{E}\left[MD_{t,H1N1=0}|SAIH_{H1N1=0},\widehat{MD_{t-1,H1N1=0}},\widehat{MD_{t-2,H1N1=0}},\widehat{MD_{t-3,H1N1=0}}\right]}{\sum_{t} \widehat{E}\left[MD_{t}|\widehat{MD_{t-1}},\widehat{MD_{t-2}},\widehat{MD_{t-3}},SAIH_{H1N1,t-1},SAIH_{H3N2,t-1},SAIH_{B,t-1},SAIH_{pH1N1,t-1}\right]}\right]$$

One to three week lagged MD autoregressive terms estimated without (numerator) and with (denominator) the influence of the influenza subtype are represented by and , respectively. In the influenza free world represented in the numerator, the MD at the previous time lag was generated by the g-formula under our modeling assumptions. The AF using the g-formula is similar to a standardized risk difference with time-varying covariates, comparing a world with no influenza to the mean of the observed world where influenza exists.[^11^](#_ENREF_11) The g-formula in the numerator takes the predicted mean from the model, sets the exposure (i.e. H1N1) to be equal to zero across time, and averages over the covariates using the distribution of covariates in an influenza-free world, as displayed below:

$\sum_{t} \sum_{x_{t-1}} E\left[MD_{t}|\overline{H1N1_{t-1}}=0,\overline{MD_{t-1}}=\overline{x_{t-1}}\right]\times\prod_{k=0}^{t-1} pr\left[MD_{k}=x_{k}|\overline{MD_{k-1}}=\overline{x_{k-1}},\overline{H1N1_{k-1}}=0\right]$

**Section S4. Testing for unmeasured confounding specific to a time-scale**

We tested for unmeasured confounding operating at two different time-scales using two versions of permutations of the data. In the first, we randomly permuted the weekly counts of MD within each winter season from October 1 to May 1 (Permutation 1). This permutation should preserve any confounding factor that is specific to particular years, hence finding that the AF in the real data is an outlier relative to this permutation indicates that there is unlikely to be confounding by year-specific common causes of MD and FLU. Secondly, we randomly permuted the MD seasons where the order of the weeks between October 1 and May 1 is preserved but the epidemic year is permuted randomly (Permutation 2). Thus the MD cases from 1998 could be aligned with FLU cases from 2004, etc. Only complete seasons were used, resulting in the inclusion of weeks from August 1, 1989 to July 31, 2009. This permutation preserves any confounders specific to a particular week of the year (e.g. the week of Christmas) that may create noncausal associations between MD and FLU; a finding that the AF in the real data is an outlier relative to this permuted distribution indicates a low risk of confounding by week-specific common causes of MD and FLU. We created 1000 permutations for permutation 1 and 10,000 for permutation two and ran the model for each permutation using the influenza model where weekly counts were lagged 1 week. The AF estimates were preserved and their distributions compared to the observed AF.

In the first permutation scenario, we found that the observed AF of 12.8% was outside the range of AF calculated from the 1000 permutations (range = -8.8 to 11.6%) (eFigure 2a) giving a *P* value <.001. When entire seasons of MD hospitalizations are randomized to different years (Permutation 2), the distribution widens, and 95% of the permuted AF lie between -3.3% and 13.7% (eFigure 2b). The observed AF of 12.8 has a *P* value relative to this permutation distribution of .035. Together, these two permutation tests found no evidence of an unobserved confounder operating either exactly on a yearly scale, or solely on a weekly scale, thus suggesting that either a causal relation exists between FLU and MD (as we hypothesize), or some confounder, whose timing varies from year to year, causes both (which is formally possible, but we know of no such candidate confounder).

**Section S5. The effect of age on the fraction of meningococcal disease attributable to influenza**

Age is an important factor that affects influenza subtype and MD serogroup susceptibility, MD carriage prevalence and disease severity.[^12-17^](#_ENREF_12) Influenza B occurs mostly in younger children and results in milder illness than influenza A/H3N2. Prevalence among age groups of MD serogroups resulting in invasive disease varies, with meningococcal serogroup B accounting for more than 50% of cases in infants. In persons aged ≥ 11 years, >75% of cases are caused by serogroups C, Y or W-135.[^18^](#_ENREF_18) The susceptibility of distinct age groups to differing MD serogroups and subtypes of influenza should affect the fraction of MD attributable to FLU.

Animal and in-vitro cell studies suggest that there is variability in the interaction between influenza types/subtypes and MD serogroups. One study found no evidence that influenza B increased epithelial adherence of meningococcus serogroup B in human nasopharyngeal cells, but influenza B could work through other mechanisms and/or with other serogroups. Influenza A might also interact differently with meningococcus serogroups, with the sialic acid containing capsular serogroups B, C and W-135 showing increased adherence to respiratory epithelial cells in the presence of influenza A neuraminidase, while the mannosamine phosphate capsular serogroup A does not.[^19^](#_ENREF_19) Given these complex interactions and the lack of biological data to suggest which interactions might be stronger, our use of age was simply a proxy to see if we could identify variability in these associations.

To explore the effect of age, we created models using the age stratified hospitalization time series for MD and all ages for FLU. Given that the majority of hospitalizations for influenza are in older adults but most transmission and mild disease occurs in children,[^20^](#_ENREF_20),[^21^](#_ENREF_21) we chose to use all ages of influenza hospitalizations modeled against different age strata of MD. We defined the age strata based on the MD incidence rates described in eSection 2: 0-4, 5-24, and greater than 25 years old. The age-stratified model is the same as the model described in eSection 3 with the exception that *E[MD_t_]* is replaced by *E[MD_t,i_]* where *i* is age stratum.

The synchrony between influenza for all ages and age-specific MD in each age group was significant. The adults 25 years and older were the most synchronized with FLU preceding MD in most seasons (ρ = 0.96 (p <.001)) (data not shown). The variability in MD was explained best in the 0-4 year old age group (R^2^ = 41%) and the model predicted the observed MD well, capturing the peaks and inter-annual variability (eFigure 3 and eTable 4). Over the seventeen year study period, influenza hospitalizations lagged one week accounted for 12.9 (95% CI 8.7-15.8), 15.5 (95% CI 10.6-19.0), and 9.2 (95% CI 4.9-12.6) percent of MD in the 0-4, 5-24, and >25 year old age groups, respectively.

The difference in the amount of observed MD that can be explained by our model between the three age groups suggest there are other risk factors not captured in our model. Adolescents have risk factors for acquiring MD that are particular to their age group, in particular kissing multiple partners and being a university student.[^16^](#_ENREF_16) It could be that there are factors that influence MD independent of influenza that are not accounted for in our analysis of the older age groups.

**Section S6. Spatial heterogeneity in the association between influenza and meningococcal disease hospitalizations**

To analyze the synchrony of peak hospitalizations across states, we abstracted 13 states that averaged more than 50 cases of MD per season: CA, FL, GA, IL, MO, NC, NJ, NY, OR, PA, TN, TX and WA. The amount of time these states contributed data to the SID varied. Given the rapid decline in MD incidence since the beginning of our study period, many of the states included had very small weekly numbers. To remove as much noise from this analysis as possible, we only included years in the analysis when the peak number of cases was at least 3.5% of the total season’s MD count. As the peak week was identified from the 5-week moving average of the time series, there were many states that had tied weeks. eTable 5 shows both the number of observations (includes all tied weeks) and the number of seasons that were included for each state. As the number of observations increased, the correlation weakened, as the peak MD weeks were spread throughout the season (eFigure 4). In states where the average weekly count of MD was larger (towards the bottom of eTable 5), the correlation strengthened as the number of tied MD weeks was smaller.

Overall, we can say that the pattern of synchrony in the timing of epidemic peaks is consistent across the US for individual states. In the four states with significant correlation coefficients, the peak in influenza hospitalizations preceded or was concurrent with MD hospitalizations most of the time. In the other states it is unclear whether the lack of synchrony was the result of small weekly counts of MD or a true lack of synchrony.

**Supporting Information**  **References**

**1.** Leader S, Kohlhase K. Respiratory syncytial virus-coded pediatric hospitalizations, 1997 to 1999. *Pediatr Infect Dis J.* Jul 2002;21(7):629-632.

**2.** Centers for Disease Control and Prevention. Overview of Influenza Surveillance in the United States. <http://www.cdc.gov/flu/weekly/fluactivity.htm>. Accessed January 8, 2010.

**3.** Centers for Disease Control and Prevention. Active Bacterial Core surveillance (ABCs) Methodology. <http://www.cdc.gov/abcs/methodology/index.html>. Accessed May 28, 2010.

**4.** Centers for Disease Control and Prevention. Active Bacterial Core Surveillance. <http://www.cdc.gov/ncidod/DBMD/abcs/team-start.htm>. Accessed December 14, 2008.

**5.** HCUP State Inpatient Databases (SID). Healthcare Cost and Utilization Project (HCUP). Agency for Healthcare Research and Quality. [www.hcup-us.ahrq.gov/sidoverview.jsp](http://www.hcup-us.ahrq.gov/sidoverview.jsp). Accessed May 5, 2008.

**6.** United States Census. Population Estimates, 2008. <http://www.census.gov/popest/estbygeo.html>. Accessed May 5, 2008.

**7.** Daly L. Simple SAS macros for the calculation of exact binomial and Poisson confidence limits. *Comput Biol Med.* Sep 1992;22(5):351-361.

**8.** Cameron AC, Trivedi PK. *Regression analysis of count data*. New York, NY: Cambridge University Press; 1998.

**9.** Abramowitz M, Stegun IA. *Handbook of Mathematical Functions: With Formulas, Graphs and Mathematical Tables*: Courier Dover Publications; 1965.

**10.** Cameron A, Windmeijer F. R-Squared measures for count data regression models with applications to health-care utilization. *Journal of Business and Economic Statistics.* 1996;14:209-220.

**11.** Robins J. Causal inference from complex longitudinal data. In: Berkane M, ed. *Latent variable modeling and applications to causality. Lecture notes in statistics 120*: Springer Verlag; 1997:69–117.

**12.** Artenstein MS, Rust JH, Jr., Hunter DH, Lamson TH, Buescher EL. Acute respiratory disease and meningococcal infection in army recruits. *JAMA.* Sep 25 1967;201(13):1004-1007.

**13.** Greenwood BM, Bradley AK, Wall RA. Meningococcal disease and season in sub-Saharan Africa. *Lancet.* Oct 12 1985;2(8459):829-830.

**14.** Moore PS, Hierholzer J, DeWitt W, et al. Respiratory viruses and mycoplasma as cofactors for epidemic group A meningococcal meningitis. *JAMA.* Sep 12 1990;264(10):1271-1275.

**15.** Stephens DS, Greenwood B, Brandtzaeg P. Epidemic meningitis, meningococcaemia, and Neisseria meningitidis. *Lancet.* Jun 30 2007;369(9580):2196-2210.

**16.** Tully J, Viner RM, Coen PG, et al. Risk and protective factors for meningococcal disease in adolescents: matched cohort study. *BMJ (Clinical research ed.).* Mar 25 2006;332(7539):445-450.

**17.** Young LS, LaForce FM, Head JJ, Feeley JC, Bennett JV. A simultaneous outbreak of meningococcal and influenza infections. *N Engl J Med.* Jul 6 1972;287(1):5-9.

**18.** Bilukha OO, Rosenstein N. Prevention and control of meningococcal disease. Recommendations of the Advisory Committee on Immunization Practices (ACIP). *MMWR Recomm Rep.* May 27 2005;54(RR-7):1-21.

**19.** Rameix-Welti MA, Zarantonelli ML, Giorgini D, et al. Influenza A virus neuraminidase enhances meningococcal adhesion to epithelial cells through interaction with sialic acid-containing meningococcal capsules. *Infect Immun.* Sep 2009;77(9):3588-3595.

**20.** Poehling KA, Edwards KM, Weinberg GA, et al. The underrecognized burden of influenza in young children. *N Engl J Med.* Jul 6 2006;355(1):31-40.

**21.** Thompson WW, Shay DK, Weintraub E, et al. Influenza-associated hospitalizations in the United States. *JAMA.* Sep 15 2004;292(11):1333-1340.
